# Matrix and Filler Recycling of Carbon and Glass Fiber-Reinforced Polymer Composites: A Review

**DOI:** 10.3390/polym13213817

**Published:** 2021-11-04

**Authors:** Roberto Scaffaro, Alberto Di Bartolo, Nadka Tz. Dintcheva

**Affiliations:** Dipartimento di Ingegneria, Università di Palermo, Viale delle Scienze, Ed. 6, 90128 Palermo, Italy; alberto.dibartolo@unipa.it

**Keywords:** recycling, FRP composites, thermoplastic polymers, thermoset polymers, glass fibers, carbon fibers

## Abstract

Fiber-reinforced polymers (FRPs) are low-density, high-performance composite materials, which find important applications in the automotive, aerospace, and energy industry, to only cite a few. With the increasing concerns about sustainability and environment risks, the problem of the recycling of such complex composite systems has been emerging in politics, industry, and academia. The issue is exacerbated by the increased use of FRPs in the automotive industry and by the expected decommissioning of airplanes and wind turbines amounting to thousands of metric tons of composite materials. Currently, the recycling of FRPs downcycles the entire composite to some form of reinforcement material (typically for cements) or degrades the polymer matrix to recover the fibers. Following the principles of sustainability, the reuse and recycling of the whole composite—fiber and polymer—should be promoted. In this review paper, we report on recent research works that achieve the recycling of both the fiber and matrix phase of FRP composites, with the polymer being either directly recovered or converted to value-added monomers and oligomers.

## 1. Introduction

Fiber-reinforced polymers are a class of composite materials in which a fiber phase is dispersed or structurally integrated in a continuous polymeric phase [1,2,3,4,5]. The main role of the polymeric matrix is to provide geometrical stability to the composite and to protect the fiber phase from the external environment. The fiber phase acts as a reinforcement; it can simply comprise of dispersed fibers with a specific aspect ratio or come in the form of woven fabric. Commonly found FRPs can contain randomly oriented fibers, oriented fibers, and continuous fibers [1,5], where it is generally observed that the same fibers will provide greater mechanical performance when oriented. Commodity thermoplastic polymers such as polypropylene (PP), polyamides (PA), high-density polyethylene (HDPE), acrylonitrile butadiene styrene (ABS), polybutylene terephthalate (PBT), and polylactide (PLA) can be reinforced to achieve engineering applications, while most thermoset FRPs are based on cured epoxy resins. By far, the main systems by market volume are glass fiber-reinforced polymers (GFRPs), which are characterized by the low production cost of the glass fibers (GFs) while maintaining satisfactory properties [6,7,8]. On the other hand, carbon fiber-reinforced polymers (CFRPs) account for a considerable monetary value of the market because of the cost inherent to the production of carbon fibers (CFs), and they achieve more advanced properties than GFRPs [7]. Since they can deliver excellent performance while maintaining low weight and good processability, FRPs find several engineering applications in the aerospace [9], automotive [10], energy [11], and construction industry [12]. The market of FRPs, and polymer composites in general, has been steadily growing in the last few years [6,13,14], and while many applications of these materials are characterized by long life cycles—e.g., their use in wind turbines and airplanes—the topic of their end-of-life (EoL) and the management of composite waste is increasingly gaining attention. The importance of composite waste management has been particularly exacerbated by the 2009 ban of composites landfilling in Germany and the increase in landfilling tax rates [13,14] as well as the approaching decommission of composite parts from wind turbines and aircrafts [15]. It can be estimated that if left unrecycled, the aircraft industry will generate 23,000 t/y (tons per year) of waste from composite materials by 2035, while the wind energy industry could generate 100,000 t/y of decommissioned blades as waste by 2030 [16]. In the automotive industry, the application of FRP composite parts has been increasingly encouraged by environmental concerns, since the lower weight of the part translates to less fuel consumption and less emissions [14,17]. Still, this increasing use will translate to larger volumes of post-consumer waste being produced on a relatively short timescale, with the risk of the benefits coming from lighter vehicles being offset by the environmental challenge of disposing of such special waste. Furthermore, the EoL directive for vehicles stipulated by the European Parliament (ELV, 2000/53/EC) states that 95% of a passenger vehicle’s mass must be reused, recycled, or recovered [14]. Therefore, the EoL of FRPs, their efficient recycling, and their place in the waste management system are topics of increasing importance [18,19,20]. Mechanical, thermal, and chemical recycling are all possible approaches for the waste management of FRPs, with the first two being much more established at the industrial level [20]. In the mechanical approach, the FRP waste is ground multiple times to yield resin-rich powders and polymer-impregnated fibers [20,21,22]. Since the grinding shortens and damages the fiber phase, the approach is more suitable to recycle cheap fibers such as GFs. Indeed, both primary and secondary recycling of GFRPs are found in industry [13,20]. In the latter case, the recovered fibers are typically reused in the production of sheet and bulk molded compounds [20,21]. The chemical approach proceeds through the solvolysis of thermoplastic FRPs or dissolution of thermoset FRPs, which enables the tertiary recycling of the polymeric matrix in the form of value-added monomers and oligomers [13,20,21]. The approach requires relatively low temperatures, and little to no grinding, therefore minimizing the damage to the fiber phase. As a result, chemical recycling is particularly researched for the recovery of expensive fibers, mostly carbon fabric from CFRPs [13,20,22]. Thermal recycling proceeds through the thermal degradation of the polymeric matrix to recover the fiber phase. The method is industrially developed for both thermoplastic and thermoset FRPs in the form of pyrolysis, fluidized bed pyrolysis, and microwave-assisted pyrolysis [13,15,20,21,22]. The degradation products are gases and oils, which can be used as fuel resulting in some form of quaternary recycling [13,20]. As a result of its energy demand, the method is more suitable to recycle expensive FRPs such as CFRPs. One drawback is that the high temperatures involved (up to 550 °C) can damage the fiber phase, which is particularly the case for GFRPs [13,21].

With the recent shift of policy toward sustainability and circularity in the European [23] and worldwide economy [24,25], it is expected that the full recycling of FRPs, with the recovery of fibers as well as value-added products from the polymer matrix, will become increasingly important. Interest on the subject is already represented by several Horizon 2020 Projects focused on the recycling of thermoset composites [26,27,28]. Academic research on the subject has also grown in the past decade, although the recovery of the fiber phase remains the main concern. Oliveux et al. [20] presented a thorough review on the recycling of FRPs, discussing different recycling techniques, companies specializing in FRP recycling, life cycle assessment of different EoL options, and reuse of recycled fibers. More recently, in 2020, Zhang et al. [22] reported on the state-of-the-art in the recycling of CFRPs. The recycling of the polymeric phase was also reviewed, although the focus was the reuse of the fiber phase, and only CF systems were discussed.

As a result of their current and future application in relevant fields such as the automotive industry, GFRPs and CFRPs will be responsible for a larger and larger part of the composite waste flow. This reality poses an environmental and economic challenge. Part of the solution rests in finding new approaches and technologies to efficiently recycle the composite materials, avoiding landfilling, extending the products’ lifespan, and valorizing the waste. While the recovery of the costly fiber phase might be prioritized, in order to maximize the sustainability of the composite, the polymeric material should also be recovered and valorized in the form of a secondary material, value-added monomers, and oligomers. Furthermore, while the production of CFs is particularly energy-intensive, the production of GFs requires similar, if not lower, energy expenditure as the polymeric matrix.

This review reports on recent academic research that has focused on the recycling of both the fiber and polymeric phase of GFRPs and CFRPs. The performance of the recovered fibers, polymers, and composites is discussed in terms of mechanical and thermal properties and compared to the original materials. Advantages and disadvantages for the different recycling approaches are taken into consideration, and so is their potential to be transferred to different recycling paradigms (e.g., primary, secondary). Future outlooks and critical aspects on which research would be required to further the full recycling of FRPs are also discussed.

## 2. Overview of Recent Research on FRP Composites

In the following paragraphs, we briefly review some of the recent topics of interest in FRP composites research. Table 1 offers some key points regarding the literature here presented, as well as the reported tensile properties, which are later used as a reference for the properties of similar recycled composites.

Various works of fundamental research have reported on the manufacturing and characterization of different FRP composites, investigating the influence of fiber concentration as well as the orientation, length, type, and chemical or other forms of pre-treatment of the fiber. For example, composites of polyamide reinforced with carbon fibers were investigated for the change in their mechanical properties with increasing weight fraction of fibers [29,30,44,45]. Generally, it is found that the tensile properties of the composites improve with the content of carbon fibers, with reported values of tensile strength and modulus of up to 100 and 20,000 MPa, respectively [30]. Several research works have also investigated the importance of fiber orientation in FRP composites, showing how the mechanical performance of the composite can significantly increase due to a considerable percentage of fibers being oriented in the direction of the load [31,33,46,47,48]. Much research effort has also gone into improving the fiber-matrix adhesion to achieve better dispersion and mechanical performance. The use of sizing and coupling agents [49,50] and different types of chemical treatments on the fiber surface [37,38,40,51] are some of the methods under study for the purpose.

More recently, the fabrication of FRP through additive manufacturing has been a highly cited research topic [52,53]. Amongst the existing additive manufacturing techniques, fused deposition modeling (FDM) is the most widespread. The polymers available to FDM are of thermoplastic nature and relatively limited in their mechanical performance, so that the parts delivered by this technique are not suitable for high-performance applications. Different research groups, in the past ten years, have considered the possibility of incorporating a reinforcing phase in the additive manufacturing process to achieve parts with better mechanical performance.

As a result of the nature of FDM, one observation shared by different works is that the fibers in the printed part are generally well oriented (along the printing direction) [33,35,54,55,56], particularly when compared to compression molded parts. This good orientation can help with obtaining strong parts. On the other hand, different authors encountered problems related to the formation of voids, porosity, and generally related to bad matrix–fiber interaction when increasing the concentration of fibers in FDM [37,46,54]. The issue is such that compression-molded parts, displaying much worse fiber orientation, can still outperform FDM-printed parts in terms of tensile strength [33].

Nonetheless, some researchers have reported on the high tensile properties of FDM-printed CFRPs. Matsuzaki et al. [54] report a tensile strength higher than 200 MPa and modulus in the order of 20 GPa. These values are found to be higher than what was observed for similar composite parts. In this case, the authors modified the nozzle of the FDM printer, adding an area in which the polymer (PLA) would melt and impregnate the CF.

The same technology was independently applied by Tian et al. [55,56] for the production of PLA CFRP composites. The authors obtained similar results as Matsuzaki et al., with values of tensile strength around 250 MPa and modulus around 20 GPa.

Recently, a large part of the literature on FRP has focused on the use of natural fibers as reinforcement [57]. The interest on this topic, which was initially justified by the low cost and availability of the natural resources, has been growing due to the recent shift in policies toward sustainable and eco-friendly production systems. Several composites based on different polymers, such as polypropylene, and natural fibers, such as jute, hemp, flax, and sisal fibers, have been already extensively studied for different applications [4,57,58,59,60,61,62,63,64]. While the tensile properties of natural fibers are significantly lower than those of carbon and glass fibers [60], their composites can still result in excellent mechanical performances [40,42,43,50,51]. Research on jute fiber-reinforced polymers reported the highest values of tensile strength and modulus near 50 and 7000 MPa, respectively [39], while the values for flax fiber-reinforced composites go up to 240 and 18,000 MPa, which rival the performance of carbon fiber-reinforced composites [43]. Furthermore, the use of natural fibers in conjugation with biodegradable polymers results in completely biodegradable composites; this is the case for polylactide-based composites [36,43].

Finally, there has been increasing academic interest in the recycling of FRPs. As the main topic of the current review, this will be discussed in detail in the following sections.

## 3. Research on the Recycling of Matrix and Fiber Phase of FRP Composites

A relatively small number of research works have focused on the recycling of polymer composite materials, i.e., a literature search for “polymer composite*” on Web of Science™ (Clarivate Analytics, Philadelphia, PA, USA) yields around 50,000 hits (30,000 ca. since 2014), as opposed to around 1000 when filtering for “recyl*” (900 ca. since 2014). The latest years have surely seen an increase in interest on the topics of plastic sustainability, plastic recycling, as well as composites recycling. As already introduced, this interest is fueled by worldwide changes in political agendas, to bring forth a more sustainable and circular economy. Polymer composites are surely no exception to this change, and their recycling has grown in importance in the past decades with their increasing use in many engineering applications [15]. It is reasonable to expect that the most socio-political pressure will be put on the recycling of GFRPs and CFRPs, because of market size and because of their increasing application.

In this section, we review a selection of recent research papers (2014 onwards) that report on the recycling of thermoplastic FRP composites (TPFRP) and thermoset FRP composites (TSFRP) reinforced with GFs and CFs. The works explore the recovery of both the fiber and matrix phase through three possibilities: the entire composite is mechanically recycled into a new composite; value-added monomers and oligomers are recovered through the chemical recycling of the composite; the polymer is designed in a way that it can be fully recovered and recycled to a virtually unchanged state.

### 3.1. Matrix and Fiber Recycling of Thermoplastic FRP Composites

The use of thermoplastic polymers in FRP composites has been growing in the past decade, particularly for applications in the transportation and energy fields [65,66]. The main advantages of thermoplastics, when compared to thermosets, is their ability to be processed and thermoformed multiple times. Their recyclability is also a desirable characteristic, which potentially enables the recycling of the whole composite by established methodologies. In the past few years, different research groups have focused their efforts on the recycling of both matrix and fibers from TPFRPs, focusing on mechanical and chemical approaches. Table 2 presents an overview of the investigated systems and recycling mechanism for the reported literature, while Table 3 summarizes the main properties of the composites and recycled composites (when available).

We identified nine papers on the recycling of GF and CF TPFRPs: four focused on mechanical recycling [56,67,68,69] and five focused on chemical approaches [14,21,70,71,72]. As summarized in Table 2, the TPFRPs under research are based on polyamide [67,68,69,70,71], polypropylene [14,68], polymethacrylates (proprietary resins branded as Elium^®^) [21,72], and polylactide [56]. Almost all the works under review present the use of CFs as the reinforcing phase [14,56,67,68,70,71,72], while GFs are in use in three of the papers [21,68,69]. It is also interesting to notice that two of the research groups made use of actual industrial waste. Offcuts, trimmings, and scrap parts from the production of commercial laminates were recycled by Kiss et al. [68], while Pietroluongo et al. [69] recycled end-of-life parts from a ten-year-old radiator.

For the sake of clarity, the research on mechanical and chemical recycling will be discussed separately in the following paragraphs.

The mechanical approaches are based on common techniques that can be encountered in primary and secondary recycling. In all works, the entire composite materials were firstly ground to obtain the feed for the manufacturing process. In Colucci et al. [67] and in Pietroluongo et al. [69], injection molding was employed to prepare the recycled composite specimens, while Kiss et al. made use of compression molding [68]. The work by Tian et al. [56] represents a particular exception of potential secondary recycling where a custom FDM printing system is employed to recycle continuous carbon fibers (CCF) with no need for grinding, which will be discussed separately. In all works, the temperatures required for the mechanical recycling fall between 200 and 300 °C, which is typical for the processing of plastics and dependent on the melting temperature (T_m_) of the polymeric phase. For example, for the recycling of ground PA CFRP through injection molding, a recycling temperature of 280 °C, fourteen degrees above the polymer T_m_, was required in Colucci et al. [67], while the recycling of PP-based systems required temperatures of 220 °C, given the lower T_m_ of the polymer [68]. While the mechanical properties of polyamides are typically higher than those of polypropylenes, the requirement of higher recycling temperature might make PA-based composite systems less appealing for mechanical recycling.

One common drawback to the mechanical recycling of FRPs is that the grinding steps shorten and damage the fibers. The shorter length, and therefore lower aspect ratio, can significantly worsen the mechanical performance of the composite since the efficiency in the load transfer between polymeric matrix and fiber phase is reduced, as predicted by the shear-lag model [73,74]. Generally, all the reviewed works observed shortening of the fibers after mechanical recycling. In Colucci et al., the virgin PA CFRP specimens displayed an average fiber length of 0.3 mm, while after one recycling step, a large number of fibers characterized by half, or less, of the original length could be observed [67]. Furthermore, the authors noticed an increase in fiber pull-out after recycling. The authors ascribed the result to the shorter average fiber length, which is known to exacerbate the pull-out effect [32,73]. Pietroluongo et al. report a noticeable drop in viscosity after recycling three times their PA GFRP composites manufactured from EoL radiator parts. In this case, grinding of the material resulted in a decrease in fiber length from 250 to as low as 50 µm [69]. The authors assumed that a fiber phase with a high enough aspect ratio would promote the formation of a rheological percolation network, therefore increasing the viscosity of the specimens. Conversely, with the drop in fiber length, the supramolecular structure would be lost, hence the lower viscosity.

**Figure 1:**
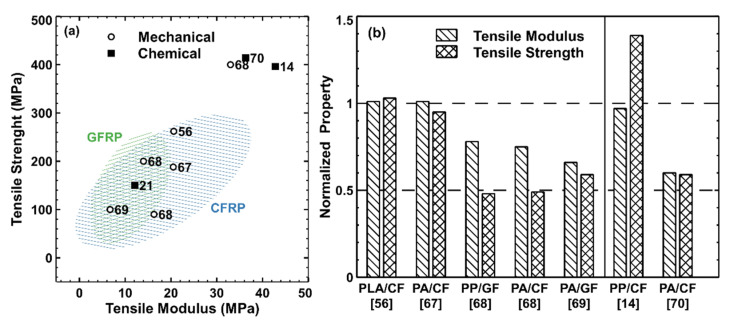
(**a**) Values of tensile strength against tensile modulus of the reviewed recycled composites. The green and blue dashed areas contain the typical values for thermoplastic GFRPs and CFRPs. (**b**) Values of tensile modulus and strength displayed by the recycled composites, normalized against the values of the virgin composites. The solid vertical line separates the values of mechanically recycled composites (left side) from the ones of chemically recycled composites.

In Figure 1a, we report the values of tensile strength against the tensile modulus of all recycled composites presented in the reviewed papers. Reference data points for pristine thermoplastic GFRPs and CFRPs are respectively contained in the green and blue dashed areas [29,30,35,44,75,76]. It can be noticed that all recycled composites have properties comparable with the reference composites found in the literature. Furthermore, in Figure 1b, we report the values of tensile strength and modulus of all recycled composites, which is normalized against the respective values of the virgin composites. The recycled composites perform relatively well, maintaining at least 50% of the original properties. One interesting characteristic to notice is that for the works in which the specimens are prepared through injection molding, the tensile properties are well maintained because the process results in a good orientation of the fibers along the flow direction [67,69]. Even in the case of Colucci et al. [67], where the material has been artificially aged, and that of Pietroluongo et al. [69], where the recycled parts are ten years old, the tensile performance of the recycled specimens is comparable to the literature. The results suggest that the recycled composites might be reused for the same applications, when the properties did not change much after recycling, or be downcycled to applications requiring lower performance. Kiss et al. further investigated the influence of the manufacturing technique on the fiber orientation and mechanical properties of their recycled laminates obtained from shredded monolithic panels reinforced by continuous fibers (either CF or GF) [68]. Injection-molded samples displayed excellent fiber alignment, while no orientation was found for the compression-molded samples. As a result, the tensile strength and modulus of the injection-molded samples were almost double those of the compression-molded ones. Still, the authors were able to obtain compression-molded panels with good tensile properties and the same flexural properties as the virgin composites by adopting a sandwich configuration in which virgin material was used as the outer layers and recycled material was used as the core layer. By doing so, 50% of the virgin material’s volume could be replaced by recycled material while maintaining good mechanical properties.

An exception to the reviewed papers on mechanical recycling of FRPs is the work presented by Tian et al. [56]. The topic of the use of CFs in conjunction with FDM 3D printing has been receiving increasing interest in the past years, which is fueled by the prospect to easily produce low-cost composite components with very specific geometries. By focusing on the recyclability of such composites, Tian et al. devised a strategy to recycle and remanufacture CFRPs by a modified FDM apparatus. The authors were able to completely recover the continuous CF and reuse it to 3D print a new generation of composites. Around 70% of the original polymer (PLA) was also recovered and reused. The recycled parts displayed comparable or better properties than the original parts, with the authors noting that the recycling process improves the impregnation of the CF. While the process might be suitable for primary or secondary recycling, the industry for 3D-printed polymer composites is still young, and therefore, it is difficult to envision the potential of this approach.

One advantage of thermoplastic FRPs over thermoset FRPs is that the absence of chemical crosslinks makes it possible for the polymer to be dissolved, enabling the chemical recycling approach with recovery of the fiber phase as well as the polymer. Typically, the complete dissolution of a thermoplastic polymer is achieved by means of suitable solvents at a given temperature. Then, the dissolved polymer can be recovered by precipitation in a non-solvent, and both chemicals can typically be recovered by distillation. In the reviewed literature, the dissolution of PP CFRP composites was carried out in boiling xylene (150 °C) [14]; PA CFRP composites were dissolved in benzyl alcohol at 160 °C [70], as well as in the proprietary solvents marketed as CreaSolv^®^, also at 160 °C [71]; GFRP and CFRP composites based on the proprietary resins Elium^®^ were respectively dissolved in chloroform [21] and either fresh Elium^®^ liquid resin or acetone [72] at room temperature. All works report high recovery of the polymeric mass, which could be reused on its own or in the preparation of a second generation of composite materials. Some of the works presented more in-depth characterizations of the recovered polymers, generally observing little degradation and small changes in molecular weight and in mechanical properties, suggesting that the recovered polymers could displace the production of virgin materials with little effect on the final product. The polypropylene precipitate recovered by Tapper et al. displayed almost unchanged mechanical properties and melting behavior with respect to the virgin polymer [14]. A small decrease in the polymer M_n_ could be observed after two recycling loops as well as worse thermal degradation behavior. The latter result was ascribed to the loss of additives after the first dissolution and therefore could be accounted for. Similar results were observed for polyamide in a continuation work by the same research group [70]. It should be noted that the decrease in molecular weight is likely to be dependent on the remanufacturing process rather than on the dissolution. Indeed, Knappich et al. [71] observed that increasing the dissolution temperature and time did not particularly affect the molecular weight of the recycled polymer.

In terms of fiber reclamation, the chemical approach delivers potentially intact fibers, since less or no grinding is required. Tapper et al. made use of 3 mm length CFs to produce discontinuous fiber-reinforced composites with PP [14] and later PA [70]. In both works, shredding was required prior to dissolution to reduce the size of the composites, although it should be noted that outside the laboratory scale, this step might not be required. The recovered fibers displayed a decrease in length to a 0.6 to 3.0 mm range, which was also ascribed to the carding performed to untangle the recovered fibers. On the other hand, the fibers were successfully manufactured into highly oriented preforms by means of a specific alignment process known as High-Performance Discontinuous Fiber method (HiPerDiF) [75]. It could be concluded that the shortening of the fibers had limited effects, since, on one hand, fiber alignment was achieved all the same, and, on the other hand, the remanufactured preforms showed better coating with matrix material. In the remaining three reviewed works, no relevant forms of size reduction were required, and the fibers maintained their length after recycling. In Knappich et al., the 80 mm length CFs in use maintained their length after recycling and displayed values of tensile strength comparable to the manufacturer details [71]. In Cousins et al., GF rovings were used to fabricate the composites, and the recovered fibers could be remanufactured into rovings with the same tensile strength and slightly lower stiffness [21]. In Gebhardt et al., the studied CFRP laminates were dissolved in acetone and yielded CF, which maintained the original fabric structure; furthermore, single fiber testing performed on the recycled CF revealed no change in ultimate tensile strength [72].

Apart from Knappich et al., all research groups proposed some form of recycling approach that, partially or completely, displaced the use of virgin materials for the manufacture of new composites. These remanufactured composites generally displayed properties comparable to reference GFRPs and CFRPs, as it can be noticed in Figure 1a for the values of tensile strength and tensile modulus reported in three of the reviewed papers [16,21,70]. The recycled PP and PA CFRP composites fabricated by Tapper et al. displayed very high tensile properties [14,70]. In this case, the composites were laminates obtained from the compression molding of the precipitated polymer and the highly aligned recycled fiber preforms, resulting in high-performance materials similarly to what was observed in Kiss et al. [68]. In the case of Tapper et al., it should be noted that no additional virgin material was required, which indicates the importance of the fiber alignment. Furthermore, the recycled PP CFRP composites displayed higher tensile strength than the original composites, which was ascribed to better fiber–matrix adhesion after recycling. On the other hand, the recycled PA CFRP composites displayed worse performance than the original materials. The authors ascribed this decrease to fiber agglomeration, breakage, and defects arising from the manufacturing. In Cousins et al., Elium^®^ GFRP spar cap parts suitable for wind turbine applications were prepared and recycled. Part of the spar caps were ground and, to this mass, a weight equivalent of recycled polymer and of PMMA (Altuglas V920) were added to obtain injection-molded tensile testing specimens, resulting in tensile strength and modulus comparable to typical GFRPs [21]. Gebhardt et al. made use of the Elium^®^ methacrylate resin to produce CFRP laminates [72]. Thermoplastic waste produced during the manufacturing process was collected and recycled by dissolving into fresh Elium^®^ resin. The authors found that around 7.5 wt % of the required virgin resin could be displaced by this recycling approach, resulting in a 40% increase in viscosity, which would still be low enough to enable the manufacturing of the specific composite parts.

To summarize, the mechanical recycling of thermoplastic FRPs shows a compromise between simplicity and the residual properties of the recyclates. Furthermore, mechanical recycling techniques are already established for both commodity plastics and composites. The main disadvantage lies in the loss of structure and length of the fibers due to the size reduction steps, which is particularly undesirable for carbon fibers. The need for different steps of milling and grinding before the remanufacturing process can also increase operational costs simply because of the energy required. Non-standard equipment, able to resist the wear caused by the fiber during grinding, might also be required. On the other hand, the recycled composites showed relatively good mechanical properties, which suggests that the materials could be downcycled to applications requiring lower performance if not reused for the original ones. At any rate, the primary recycling of composite scraps could displace the production of virgin material and eliminate the costs and fuel expenditure related to waste management, therefore being environmentally and economically benign.

The chemical dissolution is a more complex, and probably more expensive, approach because of the use of solvents and their recovery through vacuum distillation or similar processes, as well as relatively high temperatures and pressures, and long dissolution times. As a result of its cost, the approach might be justified for the recycling of the costly CFRPs. Dissolving the composite waste directly in the original polymer might be exploited in a primary recycling paradigm, although only one paper reported on such a method [72]. The main advantage of the method is that clean and undamaged fibers can be obtained as well as a reusable polymeric mass, therefore creating a closed-loop process. On the other hand, it should be noted that size reduction of the composite might be required to achieve an efficient dissolution process. This results in damage of the fiber phase and loss of its geometry greatly reducing the value of the recyclates.

### 3.2. Full Recycling of Thermoset FRP Composites

Around half the market volume of FRP composites is based on thermoset polymers [6], which are typically epoxy resins [15]. Thermoset matrices deliver excellent structural stability, resistance to chemicals and heat, high strength, high application temperature, and good weatherability, which are essential characteristics for many high-performance applications. As a result, glass, carbon, and aramid fiber-reinforced thermosets are marketed and find applications in different fields [76]. As a result of their chemically crosslinked structure, typical thermosets cannot be reprocessed by heating nor dissolved in solvents. The typical recycling approach is to thermally degrade the thermoset matrix to oils, gases, and chars to recover the valuable reinforcing phase. The degradation products are either of low value or become secondary waste; furthermore, the valuable phase might lose performance due to the harsh thermal conditions. In the past decade, many research groups have focused on the recycling of TSFRPs to recover both fiber and polymer. Here, we review a selection of recent papers where the recovery of the thermoset phase of FRPs is either demonstrated or can be legitimately inferred. It should be noted that given their high value, carbon fibers are the main topic of research. Information on the recyclable systems and methodologies is presented in Table 4, while available values for several thermal and mechanical parameters of reviewed composites are presented in Table 5.

We identified fifteen papers presenting the full recycling of thermoset FRPs; all the works make use of some form of chemical approach with two [81,82] also investigating forms of mechanical recycling. For the most part, the thermosets in use are epoxy resins, i.e., some form of cured diglycidyl ether bisphenol A (DGEBA), which is one of the most common commercial resins for FRPs manufacturing [78,79,80,81,83,84]. As anticipated, all research groups focused on carbon fiber, typically in the form of woven fabric, since its high production cost justifies the use of relatively expensive chemical recycling methodologies.

The classic approach for the chemical recycling of thermoset FRPs is the solvolysis of the polymeric matrix. The attractiveness of the method rests in the recovery of clean and, often, unaltered fibers, although the use of solvents near or above their boiling point might hinder the scalability of the processes. This method typically degrades the network completely, generating degradation products of limited value. Four of the works here reviewed showed how selective cleavage at the crosslinks can be achieved to recover value-added oligomers.

Solvents used in solvolysis processes are very often at the supercritical state, requiring precise temperature and pressure control but being able to achieve degradation of the polymeric matrix without the use of additional chemicals (e.g., catalysts) and delivering clean fibers. Okajima et al. used supercritical methanol at 285 °C and 8 MPa to break the ester bonds between the epoxy backbone and the crosslinking chains of an anhydride-cured DGEBA CFRP [77]. Since only specific bonds were broken, while the C-C bonds were preserved, the degradation products could be supplemented to fresh resin and curing agent to obtain a thermoset polymer with properties comparable to the original epoxy resin. The method proved effective in recovering fibers with the same plain fabric structure and tensile strength (3 GPa) as confirmed by tensile testing. Furthermore, composites made with fresh epoxy resin and recovered CFs displayed the same interlaminar shear strength (ILSS) as the original composites (70 MPa). While the method succeeds in delivering almost unaltered fibers and in recovering valuable polymers, there are concerns related to the human and environment risk inherent to the use of supercritical toxic fluids and to the cost of the equipment as well as the cost required to maintain the temperature and pressure conditions.

A possible alternative that also exploits the selective solvolysis of specific bonds is the use of catalyst as well as co-solvents to direct the degradation process without the need for supercritical conditions. Typically, catalysts that are able to act as Lewis acids are employed, as they can coordinate with the lone electron pair of the nitrogen at the crosslinking site. Wang et al. used an AlCl_3_ catalyst and acetic acid to recycle an amine-cured DGEBA-based CFRP [78], obtaining the selective cleavage of C-N bonds at relatively mild conditions of 180 °C. The analysis of the degradation products confirmed that oligomers from the polymer backbone could be recovered, with C-C and C-O bonds remaining intact and with N-H bonds replacing the cleaved C-N bonds. The authors suggested that the recovered products could be reused in the resin manufacturing process. The CF could be recovered after decomposition of the epoxy matrix and retained more than 97% of the virgin fiber tensile strength, although the woven structure was completely lost. While the solvents and conditions in use are economically viable and relatively safe, the inability to conserve the same fiber structure means a loss of residual value and excludes directly recycling the fiber phase for the original manufacturing process.

The same strategy of selectively breaking the C-N bonds was employed by Liu et al. [79] to recycle epoxy-based CFRP scraps from the aerospace industry. The authors used ZnCl_2_ as a catalyst, as they found it to have a strong coordination effect with the C-N bonds. Ethanol was used as solvent, and the reaction was carried out at 190 °C. The authors identified the presence of hydroxyl and amine groups in the recovered degradation products and confirmed their oligomeric nature with molecular weight of 650 g/mol. It was also shown that supplementing up to 15 wt % of the recovered oligomers to fresh resin and curing agent did not significantly alter the mechanical properties of the thermoset. The CF was recovered, and SEM and TGA analysis confirmed that the fibers were free from polymeric residues and retained their surface morphology. The use of a non-toxic solvent such as ethanol, as well as the recycling of actual industry scraps with high T_g_ of 210 °C, makes the results particularly promising.

The use of strong oxidizers can also be exploited to achieve the degradation of TSFRP, with the advantage of reducing heating and time required. For example, Das et al. [80] recycled CFRP composite waste from the aerospace industry. The recycling process was carried out over 4 h and at low temperature (65 °C), in an aqueous mixture of acetic acid and H_2_O_2_, which resulted in the formation of peracetic acid, acting as a strong oxidizer. The tradeoff for this approach comes from the need for oxidation-resistant equipment. Furthermore, the degradation in this case might be more extensive than in the previous approaches and cause loss of the valuable carbon backbone. The authors underlined that the degradation of the polymeric matrix also progressed during the distillation process required to recover the spent solvent, which represents an additional concern. Still, the authors confirmed that aliphatic and aromatic compounds were recovered from the degradation process. The products were supplemented, at 2 wt %, to fresh epoxy resin and hardener, yielding samples with tensile properties comparable to typical cured epoxy adhesives. Furthermore, the tensile properties of the recovered fibers were comparable to those of the virgin fibers.

In the past ten years, a new strategy to chemically recycle thermoset FRPs has been gaining attention. In this approach, the crosslinking chemistries are designed to yield networks that can be de-crosslinked and re-crosslinked under certain conditions. Generally, we refer to these type of polymers as covalent adaptable networks (CANs) [94,95]. These networks bear covalent bonds that can undergo bond exchange reactions (BERs)—either within the same network or reacting with a chemical—enabling them to be temporarily de-crosslinked, reshaped, and recycled. The use of such networks in the manufacturing of FRPs is particularly attractive, as the recycling process can be carried out at low or room temperature, minimizing the damage to the fiber phase. Additionally, a range of chemistries have been exploited to achieve de-crosslinking of the network under different stimuli, which makes the approach particularly versatile. Disulfide bonds, which are typically used for cured rubbers, can be exploited to achieve dynamic crosslinks, as shown in the works by de Luzuriaga et al. [81] and by Si et al. [82]. Both groups prepared CFRP composites based on disulfide-crosslinked epoxy resins. The mechanism underlying the de-crosslinking of the network is the thiol-disulfide exchange reaction between the disulfide bonds and a suitable solvent. In de Luzuriaga et al., the matrix was decomposed in a solution of 2-mercaptoethanol and DMF at room temperature over 24 h. The CF woven fabric was recovered, displaying only partial loss of the original structure. Unfortunately, the work did not characterize the degradation products nor explore their reuse. On the other hand, mechanical recycling was possible through grinding and compression molding, since the disulfide bonds undergo metathesis reactions at 200 °C, enabling a thermoplastic behavior. In Si et al., both the monomers and curing agent possessed disulfide bonds, which greatly accelerated the decomposition process. The composite was recycled in dithiothreitol at 90 °C in less than one hour. The recovered CF retained their woven structure and were reused to reinforce fresh resin, resulting in the same mechanical properties as the original composites with tensile modulus of 7 GPa and tensile strength of 320 MPa. Furthermore, the virgin matrix could be mechanically recycled by grinding and hot pressing and retained its original mechanical properties.

Transesterification reactions are among the first reactions to be exploited to achieve dynamic crosslinking. They are also particularly promising for the recycling of TSFRPs, since many of the cured epoxy resins under research and on the market (e.g., anhydride-cured resins) bear ester bonds, which can undergo transesterification in the presence of an alcohol. It should be noted that catalysts and co-solvents might also be needed to achieve efficient de-crosslinking. For example, Yu et al. [83] made use of Zn(Ac)_2_ to catalyze the decomposition of a fatty acid-cured DGEBA CFRP composite. When immersed in ethylene glycol (EG), the matrix was completely dissolved at 180 °C in 4 h, and simple evaporation of the remaining EG was sufficient to re-crosslink the thermoset. This allowed the authors to reclaim and reuse both the polymeric matrix and the CF multiple times, remanufacturing the same composites in a closed-loop process with almost no waste of epoxy resin. The same group focused their efforts on CFRPs based on an anhydride-cured DGEBA epoxy CAN [84,96]. A strong organic base, 1,5,7-triazabicyclo[0,4,4]dec-5-ene, EG, and NMP were used as catalyst, alcohol for the transesterification reaction, and co-solvent, respectively. The role of the NMP is to efficiently swell the polymeric matrix to facilitate the contact with catalyst and alcohol, therefore reducing the decomposition time. The CAN matrix was degraded in 1.5 h at 180 °C, yielding clean CFs and a polymeric solution. The recovered CF retained its woven structure as well as its tensile properties. Composites remanufactured from the reclaimed CF showed no significant change in their stress–strain curves during tensile testing, with modulus and strength around 4 GPa and 80 MPa, respectively. After evaporation of excess solvents, the polymeric residue was recovered and mixed with fresh epoxy resin to obtain newly cured thermosets. Up to 20 wt % of recycled polymer was supplemented to fresh resin before resulting in similar tensile modulus as the reference epoxy, although with half the elongation at break. Furthermore, the glass transition temperature of the same material decreased by 20 °, which was ascribed to the presence of dangling chains and branches. On the contrary, the composites recycled by Wang et al. conserved their T_g_ of more than 200 °C after three recycling cycles [85]. In their research, the authors exploited the transesterification of boronic ester linkages to recycle CFRP and GFRP composites. The authors used phenylboronic acid (PBA) to crosslink a novolac resin (NR), obtaining a network bearing boronic ester bonds as crosslinks (dubbed as PBNR). A reference matrix and reference CFRP and GFRP, obtained by curing NR with hexamethylenetetramine (HMTA, the typical curing agent for NR), were also synthetized. The PBNR composites were dissolved in ethanol at room temperature in 12 h (CFRP) and 14 h (GFRP). The recovered fibers maintained their plain sheet structure and were mostly unchanged except for some oxidation. The recovered CF and the polymer solution were also used to prepare new generations of composites. Other than conserving the high T_g_, the third-generation composites maintained their mechanical properties, with flexural modulus of 20.1 GPa, flexural strength of 380.8 Mpa, and interlaminar shear strength of 41.5 MPa. One critical issue for these composites is that curing times longer than 20 h were required, because of the very reversible nature of the crosslinking reaction. Furthermore, the times required for the complete decomposition of the composites are particularly high. These two issues are particularly critical in terms of translating the approach to a larger scale; in particular, the manufacturing time is impractical and could result in degradation of the polymeric backbone.

Transimination reactions, the BER between an imine and amine group, can also be exploited to achieve dynamic crosslinks. Interestingly, polyimines can be prepared through the reaction between aldehydes and amines, meaning that the latter can be potentially employed in the transimination reaction. This approach was taken by Taynton et al. [86], who prepared CFRP composites based on polyimine thermosets, which were cured via a triamine (tris(2-aminoethyl)amine). The resulting networks are imine-crosslinked and can be de-crosslinked upon immersion in diethylenetriamine, which is one of the comonomers used during synthesis of the polyimines. This simplified the re-crosslinking of the thermoset, as the correct amount of aldehyde monomer and curing agents can be directly added to the degradation product. It was found that a second-generation thermoset containing 33 wt % of recycled resin displayed unchanged mechanical performance with respect to the original CAN. Furthermore, the CF fabric was recovered and retained its original structure and mechanical properties. While the preparation and recycling methodology for these CFRPs are readily achieved, it should be noted that the polyimines presented in the study displayed low values of T_g_ of 18, 55, and 135 °C, which might not be suitable for high-performance applications. On the other hand, Wang et al. [87] synthetized a high-performance formyl-bearing epoxy, which was cured by a diamine (4,4′-methylenebiscyclohexanamine), resulting in a thermoset containing imine bonds in its repeating units. The CFRP based on this resin displayed T_g_ of 172 °C, T_d_ of 323 °C, tensile modulus of 35.3 GPa, and tensile strength of 763 MPa, which are all comparable to a reference DGEBA CFRP (based on DOW DER331). The thermoset also displayed CAN behavior, i.e., malleability, reprocessability, and recyclability by heating. Rather than by transimination, the CFRP composites were degraded by hydrolysis of the imine bonds in an acidic solution of methanol and water at room temperature over 15 h. Under such conditions, the hydrolysis resulted in a solution of three monomers, as confirmed by NMR analysis. The recovered CF retained its fabric structure, surface morphology, and mechanical properties, as confirmed by SEM, Raman, and tensile test analysis. In a different work [88], the authors synthetized a similar CAN and showed that the monomers produced during degradation can be effectively recovered.

Excellent thermal properties were also observed for a CFRP based on a spiro diacetal epoxy cured with isophorone diamine [89]. The thermoset was stable under neutral and basic conditions and exhibited excellent thermal stability with T_g_ of 169 °C, T_d_ of 278 °C, and 50% mass loss at 429 °C. The rigidity of the spiral diacetal confers high mechanical properties to the CFRP composites, which displayed high tensile strength and modulus of 731 Mpa and 40.0 Gpa, respectively. Such values are in the range of laminate CF composites based on other reversible networks and of high-performance epoxy FRPs, which makes the spiro diacetal material particularly promising. Furthermore, the matrix phase could be dissolved in a mildly acidic solution (HCl/acetone/water) over 30 min, yielding CF with intact morphology and unchanged mechanical properties. The degradation process exploits the cleavage of the acetal bonds. This approach has been already implemented in commercial applications such as the Connora’s Recyclamine^®^ by Connora Technologies (Hayward, CA, USA), which is a type of amine-based curing agent bearing acetal linkages that has been designed specifically for the purpose of obtaining recyclable epoxy composites. The product was used by La Rosa et al. [90] to prepare CFRPs based on a commercial bio-derived epoxy monomer, Super-Sap^®^ (Entropy Resins, Hayward, CA, USA). As discussed, the acetal bonds are labile under acidic conditions; consequently, the CFRP could be recycled in an acetic acid solution at 80 °C. The recovered carbon fibers retained their morphology, while the solid precipitate obtained from the degradation process could be converted to thermoplastic specimens exhibiting tensile strength of 55 MPa and modulus of 2.4 GPa. In continuation of this work, the authors produced a hybrid composite using the same epoxy and curing agent [91]. Hybrid composite laminates were obtained by resin infusion of different layup designs of carbon and flax fiber fabrics. The composites could be recycled by decomposition of the thermoset matrix in an acetic acid solution, the fibers were filtered out, and a polymer precipitate could be obtained by neutralizing the aqueous solution. The recovered polymer was further purified and reused as a thermoplastic feedstock in the preparation of kenaf fiber-reinforced composites through melt mixing and injection molding. The authors found that the recycled thermoplastic composite showed good mechanical properties with values of tensile modulus and strength of 2.84 Gpa and 58.87 Mpa, respectively. Furthermore, the polymer was extruded to obtain a filament that was successfully used as feed for a fused deposition modeling printer.

Bond cleavage under acidic conditions was also reported for the multiple recycling of a CFRP composite presented by Yuan et al. in 2017 [92]. The polymer thermoset was based on a poly(hexahydrotriazine) resin, which was previously reported by García et al. [97]. The hexahydrotriazine skeleton presents strong covalent bonds but can be hydrolyzed under mild acidic conditions. Furthermore, the authors made use of 2,2-Bis[4-(4-3aminophenoxy)phenyl]propane (BAPP) as monomer to obtain high thermal stability and toughness of the thermoset. Unidirectional and cross-ply CF cloth composites were prepared and tested. Both types of composites displayed high T_g_ values close to 200 °C and T_d_ up to 384 °C, the unidirectional composite displayed tensile strength of 1.8 GPa and modulus of 140 GPa, while the cross-ply composite displayed lower, but still very high, values of 741 MPa and 68 GPa, respectively. Although the hexahydrotriazine is hydrolysable under acidic condition, the thermoset showed resistance to both basic and acidic solutions. The authors observed that the resistance to acids was due to the poor wettability of the resin surface. Introducing an organic solvent (THF) with good wettability enabled the degradation of the polymeric matrix, which took 36 h at room temperature. The carbon fiber cloth could be recovered with no damage nor structure loss. The polymeric solution could also be recovered with around 90% efficiency. Recycled cross-ply composites were manufactured from both the recovered fibers and polymer and displayed almost unchanged mechanical properties over three recycling generations, with flexural modulus of 55 GPa, flexural strength of 830 MPa, and short-beam strength of 75 MPa. In summary, the reported research shows that recovery of the polymer phase in TSFRPs can be achieved by selectively cleaving specific bonds responsible for the crosslinked structure. In the case of solvolysis, the decomposition products can be reused to supplement fresh resin, or as value-added oligomers, although to a certain extent. On the other hand, in the case of CAN FRPs, the crosslinking chemistry is designed a priori to result in dynamic bonds that can be reversibly cleaved. Therefore, the thermoset can be decomposed and later reformed, virtually maintaining the exact original structure.

**Figure 2:**
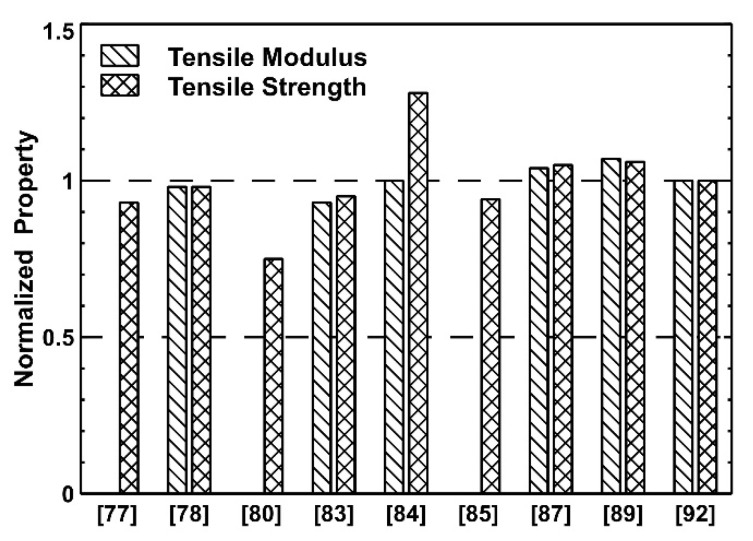
Values of tensile modulus and strength of the recycled CF, normalized against the virgin fiber values, for all references reporting such data.

In terms of fiber reclamation, most of the works showed extremely positive results with the recycling process leaving the fiber structure and properties almost unchanged. As represented in Figure 2, the values of tensile modulus and strength of the recycled fibers are often the same, or even larger, than the virgin ones. Furthermore, almost all the works reported the recycling of the polymeric phase and its use to produce new composites or polymers. In several instances, the de-crosslinking was obtained by the use of solvents, but the use of the original monomers is also possible, e.g., as shown in Taynton et al. [86]. The industrial production of composite prepregs or different components incurs costs related to the disposal of non-recyclable waste. Thermosets with reversible networks that can be de-crosslinked in an excess of monomers or comonomers might be suitable for primary recycling, since the obtained oligomeric solution can be corrected in terms of monomers and curing agent concentration and then reused.

In terms of thermal properties, most of the discussed thermoset FRPs show T_g_ lower than 200 °C, some with values around room temperature [85,88,93], which would greatly limit their application, since thermosets used in high-performance FRPs are characterized by high T_g_, around or higher than 200 °C [92]. Nonetheless, some of the works report values of T_g_ that are relatively high (170 °C) and also higher than 200 °C [81,87,89,91,94]. This shows how the appropriate chemistry can deliver a recyclable and high-performance thermoset composite.

The possibility to recover undamaged fibers and oligomers and to reprocess the materials into the same composite is the main advantage of the reviewed TSFRPs and provides a great opportunity to achieve sustainable composites. At the same time, the use of reversible chemistries in CAN FRPs might result in labile crosslinked structures with weak properties. In particular, hydrolysable chemistries might be susceptible to fast weathering, which would greatly hinder their applications. The loss of a chemically stable crosslinked structure can also result in lower mechanical properties. As a matter of fact, many of the reviewed CAN FRPs show tensile modulus and strength that are relatively low when compared to the typical properties of CF reinforced epoxy composites. The use of coatings to reduce wettability, as well as additives to increase material properties, might be possible approaches to improve the applicability of recyclable TSFRPs.

## 4. Conclusions

In the present review, we reported on recent research works that display, on an experimental scale, the possibility to recover polymers, oligomers, or monomers from the recycling of FRPs. The number of papers is limited (twenty-five in total) when compared with the research on FRPs composites and their recycling. Indeed, most research in the field is focused on the recovery of the fiber phase, with the polymeric phase being completely downcycled to fuel. With the shift of worldwide policies toward sustainability, closed-loop approaches to the production of goods and waste management will become increasingly important; we expect that this will fuel much more research about FRPs’ full recyclability. This is also to be expected in consideration of the increasing use of GFRP and CFRP composites in vehicles and other daily life products. The reviewed literature already offers valuable results in terms of the performance of multiply recycled FRPs, with CFRPs as the focus. The reviewed papers presented sufficiently exhaustive data on thermal, tensile, and flexural properties of the recycled composites. In general, it can be said that the recycled composites display good mechanical performance, i.e., comparable to those of typical virgin composites. As a result of the grinding steps, mechanical recycling approaches bear the disadvantage of damaging the fiber phase. This drawback means that their application might be more suitable for inexpensive fibers such as GFs, while the high production costs of CFs might justify and encourage the development of chemical recycling. On the other hand, the use of toxic solvents at relatively high temperature and pressures is the main disadvantage of most chemical recycling approaches. Both for the dissolution of thermoplastic FRPs, and for the de-crosslinking of CAN FRPs, this issue might raise concerns related to health and environmental hazards. The efficient reclamation of spent chemicals is also of particular interest for the sustainability of the recycling process, and most papers showed high recovery of the solvents in use. Future works and life cycle assessments should possibly focus on the sustainability of the reviewed recycling approaches, particularly in comparison with the established methodologies. More research would also need to explore the effect of weathering on CAN FRPs, particularly in the case of hydrolysable chemistries. Finally, the use of bio-derived polymers and chemicals could also be considered, which would be in line with the current growth of the bio-economy and green chemistry.

## Figures and Tables

**Table 1:** Summary of reference literature on FRPs, topics of interest, and tensile properties.

Topic	Fiber	Matrix	E, MPa	TS, MPa	Ref
Fundamental Research	Carbon	PA	1600	86	[29]
	Glass Carbon	PA	18,000 21,000	92 100	[30]
	Glass	PBT PA	8500 7000	110 125	[31]
	Glass Carbon	PP	8800 15,000	50 60	[32]
Additive Manufacturing	Carbon	ABS	1400	68	[33]
	Carbon	ABS	2500	42	[34]
	Carbon Glass Kevlar	PA	7700 3750 4370	216 206 164	[35]
Natural Fibers and Additive Manufacturing	Jute + Flax	PLA	3450	56	[36]
Natural Fibers	Jute	PP	2500	27	[37,38]
	Jute	PP	6800	44	[39]
	Jute	PP	3000	28	[40]
	Pine + Agave	HDPE	650	27	[41]
	Cellulose	PLA	3700	51	[42]
	Flax	PP PLA	17,400 18,300	215 240	[43]

**Table 2:** Summary of reviewed works on the full recycling of TPFRPs, methodologies in use, and recycling outcome.

System (Matrix/Fiber)	Recycling Mechanism	T_rec_ (°C)	Chemicals for Recycling	♲	Notes on Fibers after Recycling	Notes on Recycled Polymers and Composites	Ref
PLA/CCF (3D printed)	Mechanical: melting + FDM extrusion	240	NA	100% CCF 73% PLA	Higher tensile force due to better impregnation	Remanufactured composite retains mechanical properties	[56]
PA66/CF	Mechanical: grinding + injection molding	280	NA	Entire composite	Shorter fibers, retain adhesion to matrix	Remanufactured composite retains mechanical properties	[67]
PP/GF (Tepex^®^ scraps, offcuts) PA6/CF (Tepex^®^ scraps, offcuts)	Mechanical: grinding + compression molding	220 260	NA	Entire composite	Shorter fibers, random orientation in recycled composite	Recycled composites had poor properties, sandwich of virgin and recycled laminates showed properties comparable to virgin composites	[68]
PA66/GF (10 y/o car scraps)	Mechanical: grinding + injection molding	285	NA	Entire composite	Shorter fibers	Recycled composites showed noticeably worse properties than reference composite	[69]
PP/CF	Chemical: polymer dissolution of ground composite	≈150	Xylene Acetone	Entire composite	Shorter fibers, impregnated with PP, realigned by HiPerDiF	2nd generation remanufactured composite showed improved tensile strength	[14]
PA6/CF	Chemical: polymer dissolution of ground composite	160	Benzyl alcohol Acetone	Entire composite	Shorter fibers, realigned by HiPerDiF, agglomeration was observed	2nd generation remanufactured composite showed 40% lower tensile strength and modulus	[70]
PA6/CF	Chemical: polymer dissolution	160	Various CreaSolv^®^	Polymer is recovered	Clean, unchanged length, similar tensile strength	Remanufacturing of composite was not reported	[71]
Elium^®^/GF (methacrylate)	Chemical: polymer dissolution	RT	Chloroform Methanol	Entire composite	Rovings were recovered, slight decrease in stiffness	Recovered polymer and ground composite were reused	[21]
Elium^®^/CF (methacrylate)	Chemical: polymer dissolution	RT	Fresh monomer Acetone	Entire composite	Woven structure is retained	Recycled polymer can displace 7.5 wt % of virgin polymer for manufacturing of composite	[72]

The column identified by the recycling symbol (**♲**) in its header reports to what extent the material was recycled.

**Table 3:** Summary of mechanical and thermal properties of the reviewed TPFRP composites and recycled composites.

System (Polymers, Composites, Recyclates)	T_m_ (°C)	T_d_ (°C)	E (GPa)	TS (MPa)	ε_max_ (%)	FM (GPa)	FS (MPa)	IS (kJ/m^2^)	Ref
PLA/CCF (3D printed)			20.6	256		14.5	210	34.5	[56]
Recycled (3D printed)			20.6	262		13.3	263	38.7	
PA66/CF pristine	266	378	23.5	236	1.7				[67]
Aged composite	266	385	20.2	198	2.0				
Recycled composite	266	381	20.5	188	1.7				
Recycled PP/GF ^b^			≈9.5	≈50		≈8	≈100	≈13	[68]
Recycled PP/GF sandwich ^c^			≈14	≈200		≈17.5	≈380	≈120	
Recycled PA6/CF ^b^			≈18	≈100		≈15	≈200	≈20	
Recycled PA6/CF sandwich ^c^			≈33	≈400		≈43	≈700	≈43	
Injection molded recycled PP/GF			≈16.3	≈90					
Recycled PA66/GF (3 times)	252	351	6.7	100	4.2				[69]
PP/CF	166 ^a^		44.0	285	0.69				[14]
Recycled composite (2 times)	165 ^a^		42.8	396	0.99				
PA6/CF	218 ^a^		60.2	695	1.16				[70]
Recycled composite (2 times)	218 ^a^		36.3	414	1.12				
Ground Elium^®^/GF + recycled Elium^®^ + PMMA			12.1	150					[21]
7.5 wt % recycled Elium^®^/CF						≈14	≈500		[72]

(^a^) values for virgin polymer or recycled virgin polymer; (^b^) sheets from compression molding of ground composite scraps; (^c^) sandwich panels with recycled core and virgin outer layers; T_m_, melting temperature; T_d_, degradation temperature; E, Young’s modulus; TS, tensile strength; ε_max_, elongation at break; FM, flexural modulus; FS, flexural strength; IS, impact strength.

**Table 4:** Summary of reviewed works on the full recycling of TSFRPs, methodologies in use, and recycling outcome.

System (Matrix/Fiber)	Recycling Mechanism	T_Rec_ (°C)	Time (h)	Main Chemicals Used for Recycling	♲	Notes on Recovered Fibers	Notes on Recycled Polymers and Composites	Ref
Anhydride-cured DGEBA/CF	Supercritical methanol degradation	285	1.3	Methanol	Entire composite	Retain structure and strength	20 wt % recycled thermoset showed comparable properties	[77]
Amine-cured DGEBA/CF	Lewis-acid catalyzed cleavage of C-N bonds in acidic solution	180	6	Acetic acid AlCl_3_	Fibers	Retain fiber tensile strength Loss of structure	NR	[78]
Amine-cured epoxy/CF (Boeing waste)	Lewis-acid catalyzed cleavage of C-N bonds in alcohol	190	5	Ethanol ZnCl_2_	Entire composite	Retain surface properties	15 wt % recycled thermoset showed same properties as reference material	[79]
Aerospace CFRP waste	Peracetic acid mediated cleavage of C-N bonds	65	4	Acetic acid H_2_O_2_	Entire composite	Loss of structure	2 wt % recycled thermoset showed reference properties	[80]
Disulfide-cured DGEBA/CF	Cleavage of S-S bonds by thiol-disulfide exchange with solvent Mechanical recycling	RT	24	2-Mercapto- ethanol DMF	Fibers	Partial loss of structure	NR	[81]
	Mechanical recycling	210			Entire composite	Shorter fibers, loss of structure	Recycled composite sheets were obtained	
Disulfide-cured disulfide epoxy/CF Matrix only	Cleavage of S-S bonds by solvent activated thiol-disulfide exchange Mechanical recycling	90 180	<0.5 1	DMF Dithiothreitol	Virgin matrix only	Retain structure and mechanical properties	Virgin polymer was recycled by grinding and hot pressing, retaining its mechanical properties	[82]
Fatty acid-cured DGEBA/CF	Transesterification of ester bonds mediated by metal catalyst in alcohol	180	4	Ethylene glycol Zn(Ac)_2_	Entire composite	Retain tensile properties and structure	4th generation recycled composites showed unchanged properties	[83]
Anhydride-cured DGEBA/CF	Transesterification of ester bonds mediated by organic catalyst in alcohol/solvent	180	1.5	Ethylene glycol NMP TBD	Entire composite	Retain structure and mechanical properties	20 wt % recycled epoxy resin showed same mechanical properties as reference	[84]
Phenylboronic acid-cured novolac/CF	Transesterification of boronate linkages	RT	12	Ethanol	Entire composite	Retain overall properties	Recycled composites showed mostly unchanged properties	[85]
Triamine-cured polyimine/CF	Transimination reaction of imine bonds in the presence of excess aminic solvent	RT	NR	Diethylenetri- amine	Entire composite	Retain structure and mechanical properties	33 wt % recycled polymer added to fresh resin had same properties as the reference	[86]
Imine-bearing epoxy/CF	Hydrolysis of imine bonds in acidic solvent solution	RT	15	HCl methanol	Entire composite	Retain structure and mechanical properties	Monomers can be recovered from degradation solution (separate works)	[87,88]
Spiro diacetal epoxy/CF	Cleavage of acetal linkages in acidic solution	50	0.5	Acetone HCl	Fibers	Retain structure and mechanical properties	NR	[89]
Recyclamine^®^-cured Super- Sap^®^ epoxy/CF Similar/CF and/or flax	Cleavage of acetal linkages in acidic solution	80	1.5	Acetic acid	Entire composite	Retain surface properties	Recovered polymer with good tensile properties	[90]
	Cleavage of acetal linkages in acidic solution	80	1.5	Acetic acid	Entire composite	Retain surface properties	Recovered thermoplastic suitable for composites preparation and FDM	[91]
Polyhexahydro- triazine/CF	Hydrolysis of the triazine structure in acidic solution	RT	36	HCl THF	Entire composite	Retain structure and mechanical properties	3rd generation recycled composites showed unchanged properties	[92]
Thiocarbamate poythiourethane/CF	Dynamic exchange reaction at thiocarbamate functions	80	5	Trimethylolpropane tris(3-mercapto-propionate)	Entire composite	Retain structure and properties	Composite can be fully recycled, retains ILSS	[93]

The column identified by the recycling symbol (♲) in its header reports to what extent the material was recycled.

**Table 5:** Summary of mechanical and thermal properties of the reviewed TSFRP composites and recycled composites.

System (Polymers, Composites, Recycled Systems)	Tg (°C)	Td (°C)	E (GPa)	TS (MPa)	εmax (%)	FM (GPa)	FS (MPa)	IS (kJ/m^2^)	Ref
Amine-cured epoxy/CF	210								[79]
15 wt % recycled epoxy	169		7	80	3.2	2.4	102		
Disulfide-cured DGEBA	130	300	2.6 (E’)	88	7.1		557 ^a^	159 ^a^	[81]
Disulfide-cured DGEBA/CF							595 ^b^	194 ^b^	
Dual disulfide epoxy/CF	131	≈275	≈7	334	≈8.0				[82]
Recycled-CF composite	126		≈7	321	≈7.5				
Fatty acid-cured DGEBA/CF and 4th generation recycled composite	≈30		≈1.8	≈88	≈5.0				[83]
Anhydride-cured DGEBA/CF	157		≈4.0	≈80	≈3.5				[84]
PBA-cured novolac/CF	200					24.2	411		[85]
3rd generation recycled composite	202					20.1	381		
1-ply polyimine/CF	55		14.2	399	3.3				[86]
2-ply polyimine/CF	55		12.2	309	3.8				
1-ply polyimine/CF	135		15.5	148	1.0				
2-ply polyimine/CF	135		12.2	198	1.6				
Pristine polyimine	55		1.8	45	4.2				
33 wt % recycled polyimine			1.3	42	6.1				
Imine-bearing epoxy/CF	172	323	35.3	763	3.0				[87]
Spiro diacetal epoxy	169	278	3.13	85.0	5.10				[89]
Spiro diacetal DGEBA	132		2.53	74.0	14.2				
Spiro diacetal epoxy/CF			40.0	731	2.90				
Recyclamine^®^-cured Super- Sap^®^	102		22.9	579	3.33				[90]
epoxy/CF laminates									
Recovered thermoplastic	79.5		2.40	55.0					
Similar/FF ^c^ laminates	56.3		9.97	82.2		6.47	77.5		[91]
Similar/CF laminates	51.5		23.7	519		31.2	193		
Similar/FCF ^d^ hybrid laminates	55.6		16.3	310		6.26	90.2		
Similar/CFC ^e^ hybrid laminates	59.8		25.6	301		35.2	214		
Recovered thermoplastic			2.21	55.4					
Recovered thermoplastic/KeF ^f^			2.84	58.9					
Unidirectional CF/PHT composite	199	384	142	1806	1.4	127	1241		[92]
Cross-ply CF/PHT composite	198	376	68.3	741	1.2	54.8	829		
Recycled cross-ply CF/PHT						54.8	829		

(^a^) from manual lay-up; (^b^) from prepregs; (^c^) flax fiber; (^d^) flax-carbon-flax stacking; (^e^) carbon-flax-carbon stacking; (^f^) kenaf fiber; T_g_, glass transition temperature; T_d_, degradation temperature; E, Young’s modulus; TS, tensile strength; ε_max_, elongation at break; FM, flexural modulus; FS, flexural strength; IS, impact strength.

## Abbreviations

ABS	Acrylonitrile Butadiene Styrene
BER	Bond Exchange Reaction
CAN	Covalent Adaptable Network
CCF	Continuous Carbon Fiber
CF	Carbon Fiber
CFRP	Carbon Fiber-Reinforced Polymer
DGEBA	Diglycidyl Ether Bisphenol A
E	Elastic Modulus
ε_max_	Elongation At Break
EoL	End-Of-Life
FDM	Fused Deposition Modeling
FM	Flexural Modulus
FRP	Fiber Reinforced Polymer
FS	Flexural Strength
GF	Glass Fiber
GFRP	Glass Fiber-Reinforced Polymer
HDPE	High Density Polyethylene
IS	Impact Strength
PA	Polyamide
PBT	Polybutylene Terephthalate
PLA	Polylactide
PMMA	Poly Methyl Methacrylate
PP	Polypropylene
T_g_	Glass Transition Temperature
T_m_	Melting Temperature
TPFRP	Thermoplastic Fiber-Reinforced Polymer
T_rec_	Recycling Temperature
TS	Tensile Strength
TSFRP	Thermoset Fiber-Reinforced Polymer
